# SUMOylation of rice DELLA SLR1 modulates transcriptional responses and improves yield under salt stress

**DOI:** 10.1007/s00425-024-04565-1

**Published:** 2024-11-08

**Authors:** Telma Fernandes, Nuno M. Gonçalves, Cleverson C. Matiolli, Mafalda A. A. Rodrigues, Pedro M. Barros, M. Margarida Oliveira, Isabel A. Abreu

**Affiliations:** Instituto de Tecnologia Química E Biológica, Universidade Nova de Lisboa (ITQB NOVA), 2780-157 Oeiras, Portugal

**Keywords:** DELLA, Gibberellin, *Oryza sativa*, Productivity, Salt stress, SLR1, SUMOylation, Transcription factors

## Abstract

**Main conclusion:**

SUMOylation of SLR1 at K2 protects productivity under salt stress, possibly by modulation of SLR1 interactome.

**Abstract:**

DELLA proteins modulate GA signaling and are major regulators of plant plasticity to endure stress. DELLAs are mostly regulated at the post-translational level, and their activity relies on the interaction with upstream regulators and transcription factors (TFs). SUMOylation is a post-translational modification (PTM) capable of changing protein interaction and has been found to influence DELLA activity in Arabidopsis. We determined that SUMOylation of the single rice DELLA, SLENDER RICE1 (SLR1), occurs in a lysine residue different from the one identified in Arabidopsis REPRESSOR OF GA (RGA). Artificially increasing the SUMOylated SLR1 levels attenuated the penalty of salt stress on rice yield. Gene expression analysis revealed that the overexpression of SUMOylated SLR1 can regulate GA biosynthesis, which could partially explain the sustained productivity upon salt stress imposition. Furthermore, SLR1 SUMOylation blocked the interaction with the growth regulator YAB4, which may fine-tune *GA20ox2* expression. We also identified novel SLR1 interactors: bZIP23, bHLH089, bHLH094, and OSH1. All those interactions were impaired in the presence of SUMOylated SLR1. Mechanistically, we propose that SUMOylation of SLR1 disrupts its interaction with several transcription factors implicated in GA-dependent growth and ABA-dependent salinity tolerance to modulate downstream gene expression. We found that SLR1 SUMOylation represents a novel mechanism modulating DELLA activity, which attenuates the impact of stress on plant performance.

**Supplementary Information:**

The online version contains supplementary material available at 10.1007/s00425-024-04565-1.

## Introduction

DELLA proteins are key regulators of the gibberellin (GA) signaling pathway, exerting their role by repressing GA-regulated plant growth and developmental responses. In the presence of GA, DELLA interacts with the GA receptor, GIBBERELLIN-INSENSITIVE DWARF1 (GID1). The GA-GID1-DELLA complex increases the affinity of the SCFSLY1/GID2 E3 ligase ubiquitin complex (SLY1 in *Arabidopsis thaliana* and GID2 in rice) for DELLA, triggering DELLA polyubiquitination and consequently its degradation by the 26S proteasome. The degradation of DELLAs relieves their repression upon the GA signaling pathway by allowing the binding of transcription factors (TFs) to GA-responsive genes, leading to plant growth. When GA levels decrease, DELLA proteins accumulate and interact with key transcriptional regulators to restrain growth (Ueguchi-Tanaka et al. 2008; Hirano et al. 2010). For instance, DELLAs can sequester the transcription factors PHYTOCHROME INTERACTING FACTOR3 and 4 (PIF3 and PIF4), in addition to negatively regulating PIF3 protein abundance, mediating the crosstalk of light and GA signaling to modulate cell growth (de Lucas et al. 2008; Li et al 2016). DELLA transcriptional regulation of GA biosynthetic genes responsible for hormonal homeostasis also potentiates growth arrest. The single rice DELLA SLENDER RICE1 (SLR1) interacts with the YAB4 transcription factor to prevent the induction of *GA20ox2*, which encodes a dioxygenase that is essential for the synthesis of bioactive GA (Yang et al. 2016). In Arabidopsis, DELLA interaction with GAI-ASSOCIATED FACTOR1 (GAF1) TF triggers a negative feedback loop that activates GA biosynthesis and subsequent DELLA degradation (Fukazawa et al. 2017). The increasing number of validated DELLA interactors showcase an overlap with most hormone pathways, impacting development, fertility, and seed number (Davière and Achard 2015; Van De Velde et al. 2017).

DELLA activity is mostly modulated through post-translational modifications (PTMs), which impact its ability to interact with transcriptional regulators and modulate gene expression (Van De Velde et al. 2017). Besides ubiquitination and subsequent proteasome-mediated degradation, other PTMs influence the activity of the DELLAs in response to GA signaling. For instance, the phosphorylation by kinase EARLY FLOWERING 1 (EL1) regulates SLR1 protein stability and activity (Itoh et al. 2005; Dai and Xue 2010). A mechanism of antagonistic PTMs was proposed to regulate Arabidopsis DELLA’s interaction with specific protein partners. *O*-fucosylation by SPINDLY (SPY) promotes DELLA interaction with essential growth regulators – such as BRASSINOLE-RESISTANT1 (BZR1), PIF3, and PIF4 – leading to growth arrest (Zentella et al. 2017). Conversely, SLR1 *O*-GlcNAcylation by SECRET AGENT (SEC) blocks these interactions and promotes GA-induced growth resumption (Zentella et al. 2016; Camut et al. 2017). DELLA SUMOylation has also been indicated as a PTM controlling the stability of REPRESSOR OF GA (RGA), one of the Arabidopsis five DELLA proteins. SUMOylation is a eukaryotic ubiquitination-like process that involves the processing, activation, conjugation, and ligation of mature SMALL UBIQUITIN-LIKE MODIFIER (SUMO) to acceptor lysines (K) in the target protein (Miura et al. 2007). SUMOylation regulates protein stability, activity, and interactions with specific protein partners (Cremona et al. 2012).

SUMO attachment to RGA at lysine 65 hinders its ubiquitin-mediated proteasome degradation through blockage of GID1-RGA interaction in a GA-independent manner (Conti et al. 2014). The stabilization of Arabidopsis DELLA by SUMOylation also positively affected salt stress response, in line with observations that DELLA accumulation leads to increased salinity tolerance (Achard et al. 2006; Conti et al. 2014; Nelis et al. 2015). Yet, comprehensive knowledge regarding the impact of SUMOylation on DELLA proteins is still lacking, especially in crop species such as rice. Taking that into consideration, our goal was to identify the SUMOylation site of the single rice DELLA, SLR1. We also assessed the relevance of SUMOylation in modulating transcriptional responses under salt stress and how it impacts the interaction with transcription factors.

## Material and methods

### Phylogenetic characterization of DELLA proteins

DELLA protein sequences were retrieved from Plaza (Monocots 5.0; Dicots 5.0; https://bioinformatics.psb.ugent.be/plaza/) using SLR1 and AtRGA query terms for gene family identification (Van Bel et al. 2022). These datasets comprise 85 species for dicots and 27 species for monocots that already included species representative of the following taxa: Bryophytes (*Physcomitrium patens* and *Marchantia polymorpha*), pteridophytes (*Selaginella moellendorffii*) and gymnosperms (*Sequoiadendron giganteum*) and the *Amborella trichopoda* for it basal position in the angiosperm lineage and *Spirodela polyrhiza* and *Zoostera marina* for their basal position in the monocot lineage. To add more protein sequences into the pteridophytes group, we retrieved *Ceratopteris richardii* DELLA sequences from Phytozome (https://phytozome-next.jgi.doe.gov/). We also added *Thuja plicata* and *Taxus chinensis* DELLA sequences into the gymnosperm group from Phytozome and NCBI genome (https://www.ncbi.nlm.nih.gov/genome/), respectively (Goodstein et al. 2012; Van Bel et al. 2022). The DELLA-like sequences lacking the N-terminal DELLA regulatory domain from angiosperms and gymnosperms (DELLA 3) were removed.

Phylogenetic analysis was performed using Cipres (http://www.phylo.org) (Miller et al. 2015). First, sequences were aligned with the MAFFT algorithm, and maximum likelihood trees were inferred using the PROTCAT model, JTT matrix, and 1000 bootstrap replications in RaxML v8.2.12. The consensus-rooted tree was visualized and annotated using the FigTree software (v1.4.4, http://tree.bio.ed.ac.uk/software/figtree/).

### SUMOylation prediction sites

To predict the SUMOylation sites of the DELLA proteins, the sequences were submitted to GPS-SUMO 2.0 (https://sumo.biocuckoo.cn/) (Zhao et al. 2014) (Table S1). Subsequently, only sites displaying a high probability of SUMOylation were considered for the phylogenetic analysis.

### Plant material and growth conditions

The rice variety used in this study was *Oryza sativa* L. ssp. *japonica* cv. Nipponbare. Plants were grown under natural long-day conditions at the greenhouse during the rice growing season (May–August) at ITQB NOVA, Portugal (Lat. 38.696189, Long. –9.320762), and in controlled-growth chambers conditions during the winter and for assays performed in this study (12 h light at 28 °C/12 h dark at 24 °C, 400 μmol m^−2^ s^−1^) with a relative humidity of 60%.

### SLR1 cloning and vector construction

The SLR1 coding sequence was amplified using a high-fidelity PCR (Phusion DNA Polymerase; NEB) and cloned into the pJET 1.2/blunt-end cloning vector (Thermo Fisher Scientific), pET28a expression vector (Novagen) and pDONR221 (Thermo Fisher Scientific). For the generation of rice transgenic lines overexpressing SLR1, the SLR1 coding sequence was amplified from pJET-SLR1 using OsSLR1-GW-Fw and OsSLR1-GW-Fw primers to insert the attB1 and attB2 recombination sites. The amplified PCR product was recombined into the pDONR221 (Thermo Fisher Scientific). The SUMO1SLR1 gene fusion was generated using high-fidelity PCR (Phusion DNA Polymerase; NEB) as described in Atanassov et al. (2009). Primers overlapping both the OsSUMO1 3’-end and OsSLR1 5’-end OL-SLR1 + SUMO1-3’-Fw and OL SUMO1 + SLR1-5’-Rv were used in combination with OsSLR1-Gw-Rv and OsSUMO1-GW-Fw, respectively, to generate the fragments to be fused. In the next step, the PCR products were purified and used in the fusion PCR reaction containing the OsSUMO1-GW-Fw and OsSLR1-Gw-Rv primers for the final amplification of the final fusion product (Table S2). The gene fusions were confirmed by Sanger sequencing. To obtain rice transgenic plant lines overexpressing SLR1 (SLR1-OX) and SLR1 fused to SUMO1 (SUMO1SLR1-OX), pDONR221 containing the inserts described above was used as the entry vector for the pANIC6B destination vector (Mann et al. 2011). The expression of SLR1 and SUMO1SLR1 is controlled by the maize *ZmUbi1* promoter (Fig. 3b).

### Site-directed mutagenesis

To produce truncated recombinant SLR1 protein (SLR1t) containing the leading 190 amino acid residues of SLR1, a stop codon was introduced by site-directed mutagenesis (SDM) in pET28a-SLR1 using *Pfu*Turbo DNA polymerase (Agilent) and the primers (SLR1t-STOP-Fw and SLR1t-STOP-Rv) designed with QuickChangeII online tool (Agilent) (Table S2). Unmodified plasmids were digested with methylation-sensitive enzyme *Dpn*I (New England Biolabs). The mutated plasmids were transformed in *E. coli* DH5α and the mutagenized site was confirmed by sequencing. Mutated pET28a coding the truncated SLR1 isoform (SLR1t) was subjected to further mutagenesis procedures to perform the amino acid substitutions K2R and K60R (primers SLR1t-K2R-Fw, SLR1t-K2R-Rv, SLR1t-K60R-Fw and SLR1t-K60R-Rv, all listed in Table S2) following the same protocol.

### Recombinant protein production and purification

pET28a vectors containing the full-length SLR1, N-terminus truncated SLR1 (SLR1t), SLR1t (K2R) and SLR1t (K60R) were transformed into *E. coli* strain BL21 Star (DE3, Sigma-Aldrich) for recombinant protein expression. Cells were grown at 37 °C until OD_600_ = 0.6, and protein production was induced by the addition of 200 μM isopropyl-d-1-thiogalactopyranoside (IPTG). After three hours, the cells were harvested by centrifugation in a J2MI centrifuge (Beckman) at 7669 g for 20 min at 4 °C. The cells were resuspended in lysis buffer (20 mM phosphate buffer, pH 7.5, 500 mM NaCl, 10 mM imidazole) containing 5 µg/mL of DNAse, 50 µg/mL of lysozyme, 100 mM PMSF, and 5 mM MgCl_2_. After freezing at -20 °C and thawing, extracts were loaded into a HisTrap chelating column (Amersham Biosciences), and proteins eluted in phosphate buffer containing 500 mM imidazole, following buffer exchange dialysis (20 mM phosphate buffer, 150 mM NaCl, 1 M DTT, 2% glycerol), for increased protein stability. Recombinant proteins were analyzed by Western blot using a custom-made SLR1-specific polyclonal antibody.

### In vitro SUMOylation assays and ULP1 treatment

The in vitro SUMOylation assays were performed in 20 μL of SUMOylation reaction buffer (Werner et al. 2009) containing 3 µg of SLR1 recombinant protein, 1 μg of E1-activating enzyme (OsSAE1/OsSAE2), 0.8 μg of E2-conjugating enzyme (OsSCE1a), 0.5 μg of OsSUMO1/2, 0.4 units of creatine phosphokinase, and 10 mM creatine phosphate. Reactions were initiated by adding 1 mM ATP and then incubated at 30 °C for 1 h to 3 h. To confirm SUMOylation specificity, 3 μg of purified SUMO protease ULP1 from *Saccharomyces cerevisiae* (Li and Hochstrasser 2003) was used for deSUMOylation analysis. SUMO conjugates were analyzed by Western blot probed with custom anti-SLR1 antibody (1:5 000) and anti-rabbit HRP conjugate antibody (A16096 Novex; 1:20 000).

### Gibberellic acid (GA_***3***_), paclobutrazol (PAC), and salt stress assays

To assess the SLR1 protein levels in 14-day-old seedlings from shoots after GA_3_, PAC, and salt stress, dehusked rice seeds were surface sterilized with 70% ethanol for 10 min and further sterilized with 25% bleach (commercial bleach) for 15 min, then washed five times in sterile water. The seeds were placed in hydroponics with half-strength Murashige & Skoog medium and MES buffer (250 mg/L; Duchefa) with pH adjusted to 5.2. Seedlings were grown for 7 days, and the hydroponics solution was replaced with a new one supplemented with 10 µM GA_3_, 0.1 µM PAC, or ethanol (mock). After 7 days of treatment, plant shoots were collected and frozen in liquid nitrogen until SLR1 detection.

Salt stress was applied 14 days after germination, by replacing the the hydroponics solution with a new solution supplemented with 120 mM NaCl (Salt). Electric conductivity was set and maintained at 12 dS/m with a sodium chloride solution for an 8-h treatment period. A control group was obtained by renewing the hydroponics solution. After 8 h, plant shoots were collected from Salt and Control, frozen in liquid nitrogen, and stored at − 80 °C, until SLR1 detection.

For the salt assays using SLR1-OX and SUMO1SLR1-OX transgenic lines, heterozygous transgenic seeds were germinated and selected through screening for hygromycin resistance and GUS staining. Seedlings were transferred to hydroponics for salinity resistance screening (Fernandes et al. 2022) with half-strength Murashige & Skoog medium and MES buffer (250 mg/L; Duchefa) with pH adjusted to 5.2. Seedlings were grown for 14 days before the salt stress assay. Then, the seedlings were exposed to salt stress, with electric conductivity set and maintained at 12 dS/m with sodium chloride solution for the extension of the stress treatment (120 mM). After 8 days, the seedlings were transferred to the control medium for recovery. Stress severity (SES score) – 1 for lower severity and 9 for higher severity—was evaluated as set by IRRI (Gregorio et al. 1997) at 6 and 8 days after stress and 8 days after recovery. Samples for transcriptomic analysis – 4 replicates of a pool of 3 plants—were collected 8 h after the initiation of the salt stress, corresponding to 10 h after the lights went on, for both control and salt treatments, and frozen immediately in liquid nitrogen for grinding and analysis.

For the booting stage assay, previously germinated and selected heterozygous transgenic seeds were sown in plastic pots (23 cm in height, 28 cm in diameter) containing a 10 kg homogeneous mixture of 2:2:1 (by vol.) soil, peat moss, and vermiculite and placed into trays in a greenhouse. The experiment was set up in the greenhouse from June to September 2020, according to IRRI standard screening procedures (Gregorio et al. 1997). Panicle initiation was evaluated by the emergence of the flag leaf with a distance between 1 and 2 cm to the adjacent leaf and salinity was applied individually for each genotype at that point. Electric conductivity was set and maintained at 8 dS/m with sea salt for the extension of the life cycle. Upon complete maturation of the seed, agronomical parameters were measured (plant height, tillering, and panicle length), and panicles were collected for further characterization of fertility and productivity (weight of 1000 seeds as measured in a Pfeuffer seed counter). The significance of statistics was asserted by t-test with Holm-Sidak method correction for multiple comparisons and a *P* < 0.05 threshold.

### SLR1 protein extraction, detection, and quantification analysis

Plant shoot samples were ground in liquid nitrogen and 100 mg of ground tissue was used for protein extraction with denaturing modified TCA/acetone method (Luís et al. 2016), followed by resuspension in lysis buffer (7 M urea, 2 M thiourea, 30 mM Tris, 4% (w/v) CHAPS, 4% (v/v) complete protease inhibitor cocktail EDTA-Free (Sigma-Aldrich). Protein quantification was performed with a 2-D Quant kit (GE Healthcare) following the manufacturer’s instructions and 5 µg of protein extract was loaded for the Western-blot analysis. Anti-SLR1 immunoblots were performed in a Mini-Protean II System (Biorad), using custom-made anti-SLR1 (1:5 000) and anti-rabbit HRP conjugate antibody (A16096 Novex; 1:20 000) in 5% non-fat dry milk in TBS 1X.

Coomassie Brilliant Blue (CBB) staining for Rubisco (RbcL) was used as a loading control, and protein quantification of Western-blot bands was performed using the software platform Fiji (ImageJ) (Schindelin et al. 2012).

### Rice transformation

Embryogenic calli induced from mature seeds were used for rice transformation according to (Almadanim et al. 2017), and selection for transformants asserted by hygromycin resistance and standard GUS-staining with X-Gluc solution (Roth) of a section of seminal roots. Proteins were extracted, detected, and quantified using a modified TCA/acetone method (Luís et al. 2016). Due to growth arrest phenotype and fertility decrease in homozygous lines of SUMO1SLR1-OX lines, all assays continued with positively selected heterozygous plants. Moreover, lines #2 for both SUMO1SLR1-OX and SLR1-OX were selected for further analysis at the rice booting stage and transcriptomic evaluation according to non-SUMOylated SLR1 levels to avoid DELLA pleiotropic effects (Davière and Achard 2015).

### RNA extraction and analysis

RNA was extracted from a total of 100 mg of ground shoot sample with a Direct-zol kit (Zymo Research) according to the manufacturer’s protocol with in-column DNAse treatment. The first-strand cDNA was synthesized from 2 μg total RNA with an anchored-oligo (dT)18 primer, according to the instructions from Transcriptor High Fidelity cDNA Synthesis Kit (Roche). Reverse transcription quantitative PCR (RT- qPCR) analysis was performed using a LightCycler 480 system (Roche) and the SYBR Green I Master mix (Roche). The RT-qPCR reactions were carried out with the primers listed in Table S2. PCR running conditions were: one cycle at 95 °C for 5 min and 45 cycles of amplification at 95 °C for 10 s, 56–60 °C for 10 s and 72 °C/10 s. The CT values were calculated using three technical replicates, and gene expression was assessed relative to described internal transcript controls, rice ubiquitin conjugase 2 (*OsUBC2*) and ubiquitin 10 (*OsUBQ10*) (Moraes et al. 2015; Pabuayon et al. 2016).

### RNA-seq and data analysis

Shoots from 14-day-old seedlings of Nipponbare, SLR1-OX #2, and SUMO1SLR1-OX #2 in control and subjected to salt stress for 8 h (120 mM NaCl) were collected and frozen in liquid nitrogen. RNA was extracted from 100 mg of ground shoot sample with a Direct-zolTM kit (Zymo Research) according to the manufacturer’s protocol with in-column DNAse treatment. Small RNAs were cleaned using the RNeasy spin column from RNeasy Mini Kit (Qiagen) following RNA Cleanup protocol RNA quality was assessed by Fragment Analyzer (Thermo Fisher Scientific). A total of 18 cDNA libraries were prepared, using three biological replicates for each genotype (SLR1-OX, SUMO1SLR1-OX, Nipponbare) and each condition (mock and salt). The cDNA libraries were obtained from 10 ng total RNA and using an optimized SMARTSeq2 protocol. The samples were sequenced on Illumina NextSeq 500 instrument (High Output, 75 cycles, Single-end). Libraries preparation and sequencing were performed at the IGC Genomics Unit (Oeiras, Portugal).

The reads were subjected to quality check in FastQC Toolkit for data analysis. Adaptor and low-quality reads were further processed by trimmomatic software, followed by genome annotation to the *Oryza sativa* spp. *japonica* reference genome (IRGSP-1.0) (Ensembl Plant v56) by STAR. Differential expression analysis was performed using the DESeq2 R package. *P*-value < 0.05 and |log2 fold change|> 1.5 was set as the threshold for significantly different expressions (Table S3). Gene ontology (GO) enrichment analysis of differentially expressed genes was implemented by g:Profiler (Kolberg et al. 2023). GO terms with corrected *P*-value < 0.05 were considered significantly enriched.

### Promoter analysis and interaction validation by Y2H and BiFC

The 1000 bp promoter regions of selected DEGs were downloaded from the Rice Annotation Project Database (RAP-DB) (Sakai et al. 2013) and submitted to PlantPAN 2.0 (Chow et al. 2016) to find *cis*-elements and predict transcription factor binding sites (TFBS). Gene Group Analysis was used to search for common (90%) TFBSs present in DEGs promoters and corresponding TFs. Rice TFs were examined individually for experimentally verified data concerning the interaction with binding sites and the listing of downstream target genes.

Interactions between SLR1 and candidate target TFs predicted from the previous analysis were first assessed by yeast-two hybrid (Y2H) assays. The coding sequences of SLR1 and SUMO1(_GG-AA_)-SLR1 were cloned into the pGADT7 vector (Stratagene) while bHLH089, bHLH094, bZIP23, IDEF1, OSH1, OSBZ8, PCF1 and YAB4 were cloned into the pGBKT7 (Stratagene) vector. These constructs were transformed into *Saccharomyces cerevisiae* strain Y2HGold (Clontech) by applying the lithium acetate method. Yeast-transformed cells were plated on a synthetic defined (SD) medium lacking leucine and tryptophan for plasmid transformation control, and in SD lacking leucine (-Leu), tryptophan (-Trip), adenine (-Ade), and histidine (-His) for interaction screening. The interactions were evaluated in three individual colonies transformed with both plasmids. pAD-WT and pBD-WT from the HybriZAP 2.1 kit (Stratagene) were used to control positive interaction (C +). pGBKT7 and pGADT7 empty vectors were also co-transformed with pGADT7-SLR1, pGADT7-SUMO1(_GG-AA_)::SLR1 and pGBKT7-bHLH089, pGBKT7-bHLH094, pGBKT7-bZIP23, pGBKT7-IDEF1, pGBKT7-OSH1, pGBKT7-OSBZ8, pGBKT7-PCF1 and pGBKT7-YAB4, respectively, as negative controls.

For bimolecular fluorescence complementation (BiFC), SLR1, SUMO1(_GG-AA_)-SLR1 were cloned in YFN43, and bHLH089, bHLH094, bZIP23, IDEF1, OSH1, OSBZ8, PCF1 and YAB4 were cloned into YFC43. *Nicotiana benthamiana* leaves were co-infiltrated with *Agrobacterium tumefaciens* strain EH105, harboring the appropriate vectors. Fluorescence was visualized using the Leica DM6 B fluorescence microscope (40x) with an L5 filter cube (Leica) 48-72 h following infiltration. As negative control, YFN43 (empty vector) was also co-infiltrated with YFC43-bHLH089, YFC43-bHLH094, YFC43-bZIP23, YFC43-IDEF1, YFC43-OSH1, YFC43-OSBZ8, YFC43-PCF1 and YFC43-YAB4. *Agrobacterium* expressing the viral silencing suppressor P19 was included in all infiltrations to increase the expression in *N. benthamiana* leaves. The images were processed using the software platform Fiji (ImageJ) (Schindelin et al. 2012).

### Accession numbers

Sequence data from this article can be found in the Rice Annotation Project Database (RAP-DB) under the following accession numbers: SLR1 (Os03g0707600), SUMO1 (Os01g0918300), SUMO2 (Os01g0918200), RGA (At2g01570), CPS4 (Os04g0178300), KSL7 (Os02g0570400), CYP701A8 (Os06g0569500), CYP99A3 (Os04g0178400), CYP76M7 (OS02G0569900), CYP76M8 (Os02g0569400), CYP71Z7 (Os02g0570700), CKX2 (Os01g0197700), CKX5 (Os01g0775400), LOX2 (Os08g0509100), CHT3 (Os06g0726100), CHT8 (Os10g054290), CHT11 (Os03g0132900), GA20ox2 (Os01g0883800), GA2ox3 (Os01g0757200), PCF1 (Os04g0194600), bHLH089 (Os03g0802900), bHLH094 (Os07g0193800), bZIP23 (Os02g0766700), OSH1 (Os03g0727000), OSBZ8 (Os01g0658900) IDEF1 (Os11g0175700), YAB4 (Os02g0643200), RAB21 (Os11g0454300), P5CS (Os05g0455500), OsUBC2 (Os02g0634800) and OsUBQ10 (Os06g0681400).

## Results

### In vitro* SUMOylation of SLR1 takes place at lysine 2*

**Fig. 1:**
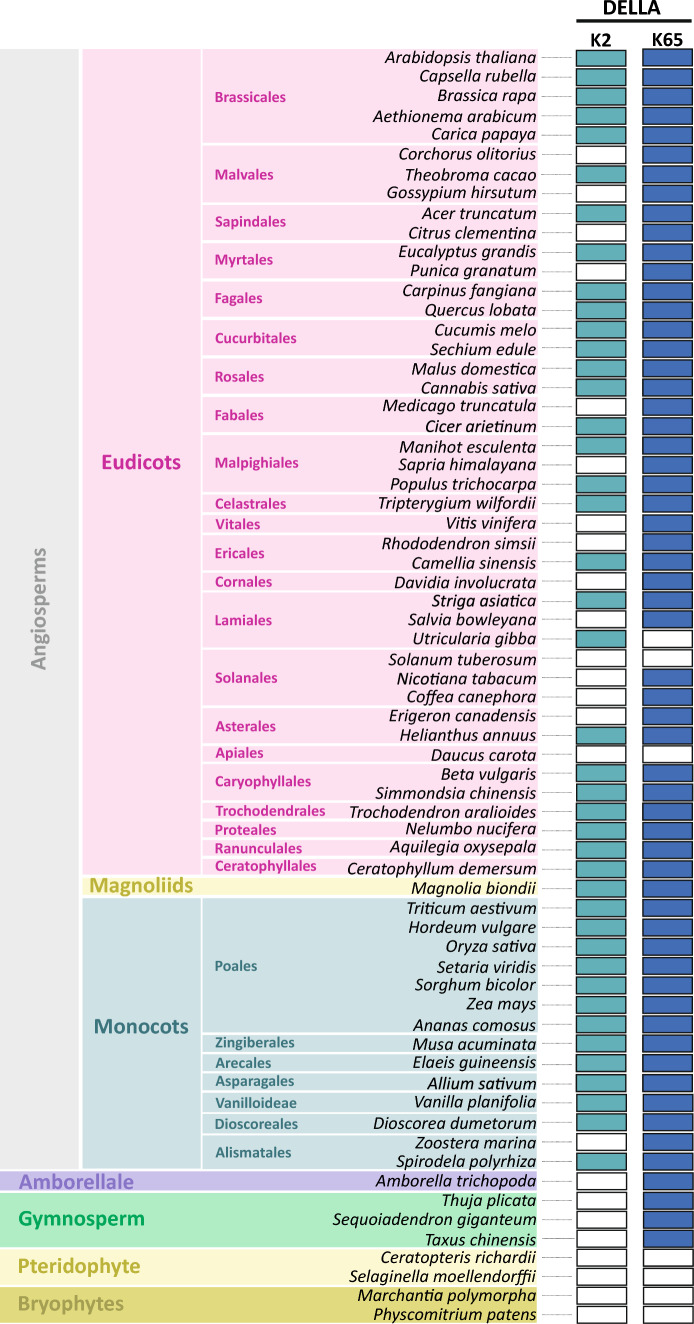
Representation of the presence of SUMOylated lysines (K2 and K65) in several plant DELLA proteins, following a phylogenetic analysis of DELLA proteins (Fig. S1). SUMOylation motifs focusing on lysine 2 and the correspondent lysine 65 were investigated using GPS-SUMO 2.0 at the DELLA domain. Blue and white rectangles display the presence or absence of SUMOylation on the investigated lysines, respectively

SUMOylation of lysine 65 (K65) in Arabidopsis DELLA proteins RGA and GAI affects their stability independently of the plant hormone GA (Conti et al. 2014). To explore whether a similar modification occurs in the rice DELLA protein SLR1, we used an in silico prediction using GPS-SUMO 2.0 (Zhao et al. 2014) to predict SUMOylation motifs on RGA and SLR1. We then selected only high-probability SUMOylation motifs on the DELLA domain (N-terminal) and combined the SUMOylation prediction with a phylogenic analysis targeting DELLAs from 119 species representing all major plant taxa (Fig. 1, Fig. S1, Table S1).

**Fig. 2:**
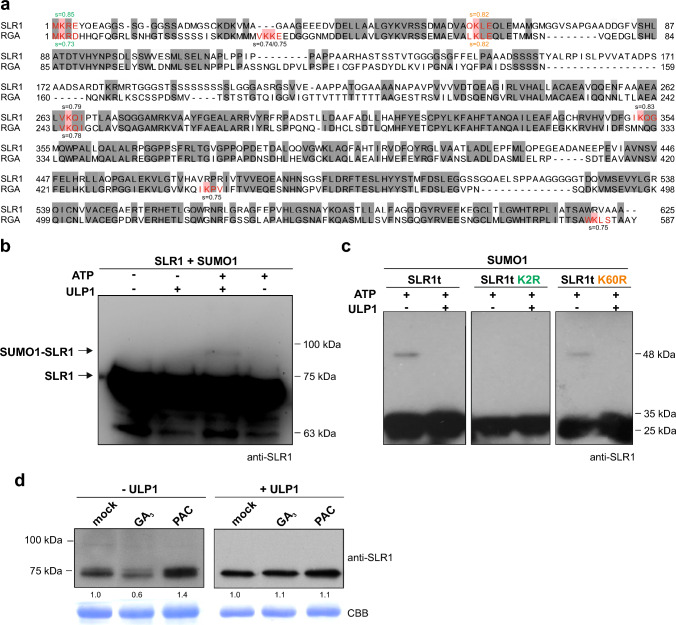
SLR1 is SUMOylated in a novel SUMO-conjugation canonical motif. **a** In silico prediction of SLR1 SUMOylation motifs scored by GPS-SUMO2 and comparison with AtRGA. K2 and K60 are marked in green and orange, respectively. **b** In vitro SUMOylation assays using rice SUMOylation machinery (SCE1, SAE1/2, and SUMO1) with recombinant full SLR1 (~ 65 kDa) and SLR1t (~ 19 kDa) proteins, treated with ULP1 SUMO protease, as indicated. SLR1 and SLR1t were detected by immunoblotting using a custom-made anti-SLR1 antibody, where **b** corresponds to the full-length SLR1, and **c** to the SLR1 N-terminal truncated (SLR1t) coding the leading 190 first SLR1 residues, as well as the mutated isoforms SLR1t K2R and SLR1t K60R. **d** Shoot protein extracts of 14-day-old Nipponbare plants subjected to ethanol (mock), GA_3_ (10 µM) and PAC (0.1 µM), immunoblotted with a custom-made anti-SLR1 antibody. ULP1 treatment (right panel). Coomassie Brilliant Blue (CBB) staining for Rubisco (RbcL) was used as a loading control. The band density was measured with ImageJ and the quantification showed beneath the WB analysis

The combined analysis revealed that no predicted SUMOylation sites were identified on the DELLA domain in early land plants, including bryophytes and lycophytes, as well as in the fern *Ceratopteris richardi.* Among the gymnosperms targeted in the study, all displayed a high probability SUMOylation site on the lysine residue corresponding to K65 in Arabidopsis RGA (hereafter referred to as K65), although within a non-canonical SUMOylation motif (QKLE). Regarding flowering plants, all monocots, except *Zoostera marina,* displayed a high probability SUMOylation site on K2 and K65, while 67% of the eudicots have at least one DELLA sequence featuring both or either K2 or the correspondent K65 SUMOylation sites. The predicted SUMOylation sites on K2 were consistent among the taxa displaying a canonical SUMOylation site (MKRE), while K65 was included in mainly two motifs: QKLEQ and LQLE (non-canonical and canonical SUMOylation sites, respectively). In sum, our in silico predictions show that DELLAs from monocots and eudicots have two high probability SUMOylation sites (K2 and K65) (Fig. 1). For SLR1 and RGA, specifically, both share a high probability SUMOylation sites at K2 and K65 (K60 in SLR1) and several putative SUMOylation motifs on the DELLA GRAS domain (C-terminal) (Fig. 2a).

To assess the SLR1 SUMOylation, we performed an in vitro SUMOylation assay targeting full-length recombinant SLR1. Upon in vitro SUMOylation, we observed the appearance of an additional SLR1 band, corresponding to a *ca.* 20 kDa mobility shift of the non-modified SLR1 (Fig. 2b), suggesting SLR1 SUMOylation. This band disappeared upon treatment with recombinant SUMO protease (ULP1) that cleaves SUMO from its conjugates (Li and Hochstrasser 2003), confirming that the observed mobility shift was caused by the attached SUMO (Fig. 2b). To discard the putative contribution of the predicted SUMOylation motifs in the GRAS domain, we produced a truncated form of SLR1, including only the DELLA regulatory domain (tSLR1). Again, we observed a mobility shift of SLR1 consistent with SUMOylation in the presence of SUMO1 and SUMO2 (Fig. 2c, Fig. S2a, b).

To discriminate SUMOylation at K2 and K60 SLR1 residues, we performed directed mutagenesis, replacing these lysine residues with arginine residues (K2R and K60R) in the SLR1 truncated form. The mutated SLR1t K60R isoform retained the ability to be SUMOylated, contrary to SLR1t K2R (Fig. 2c). The result shows that K2 is the preferential SUMOylated residue in SLR1 in our in vitro conditions.

To demonstrate the in vivo SUMOylation of SLR1, we grew WT plants under different treatments (mock, GA_3,_ and PAC) and assessed SLR1 levels by Western blot before and after ULP1 treatment. The ULP1 treatment allows for indirect detection of previously SUMOylated SLR1. Samples not treated with ULP treatment showed the expected profile, with SLR1 degradation in the presence of GA_3_ and accumulation under PAC (an inhibitor of GA biosynthesis) treatment. Protein extracts after the ULP1 treatment showed similar intensities of the SLR1 band among the treatments, suggesting an input of SLR1 resulting from its deSUMOylation (Fig. 2d). This result confirms the in silico predictions and is in line with in vitro analyses, indicating that SLR1 can be SUMOylated in vivo. Our results also suggest that SLR1 mono-SUMOylation is much less abundant than other forms of SUMOylation since an SLR1 band with the expected mobility shift was not detected for mono-SUMOylation (Fig. 2d).

### Overexpression of SUMOylated SLR1 increases salt tolerance

**Fig. 3:**
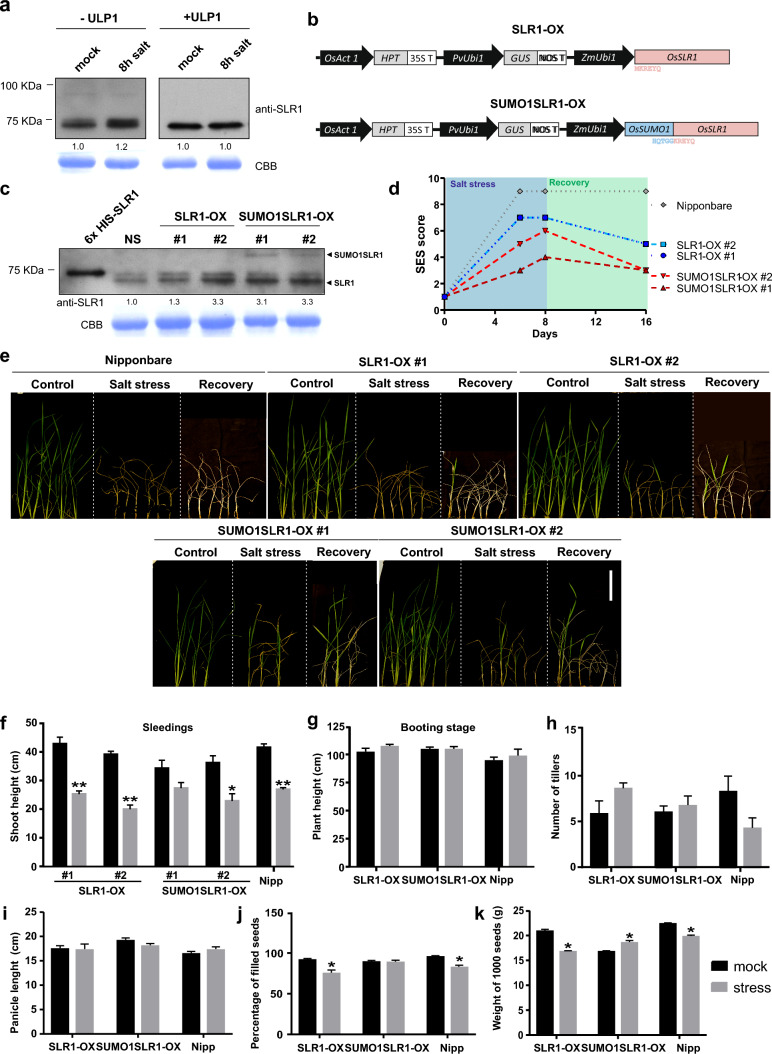
SLR1 SUMOylation confers salt tolerance at the seedling stage and reduces the impact of salt stress on yield. **a** Accumulation of SLR1 under salt stress. Shoot protein extracts of 14-day-old Nipponbare plants subjected to 120 mM of NaCl for 8 h. Immunoblotting with a custom-made anti-SLR1 antibody. ULP1 treatment (right panel). Coomassie Brilliant Blue (CBB) staining for Rubisco (RbcL) was used as a loading control. The band density was measured with ImageJ and the quantification showed beneath the WB analysis. **b** Schematic illustration of gene constructs used to generate rice SLR1-OX and SUMO1SLR1-OX transgenic lines. *Zea mays* Ubi1 (ZmUbi1) promoter drives the expression of both SLR1 (SLR1-OX) and SLR1-SUMO1 gene fusion (SUMO1SLR1-OX). **c** SLR1 and SUMO1SLR1 protein accumulation in T1 independent transgenic lines (#1 and #2) of SUMO1SLR1-OX and SLR1-OX, negative segregant (NS) and SLR1 recombinant protein used as positive control. Immunoblot using a custom-made anti-SLR1 antibody. **d** Stress severity as evaluated by IRRI salinity trial SES score for all lines in 6 and 8 days of stress and after 8 days of recovery period in 14-day-old seedlings (*n* = 6 and *n* = 3 for SUMO1SLR1-OX #1) **e** Representative plants depicting the differences in shoots of 14-day-old Nipponbare, SLR1-OX, and SUMO1SLR1-OX plants after 8 days of salinity imposition with 120 mM NaCl and a recovery period of 8 days. Scale bar = 10 cm. **f** Shoot height seedlings measurements for all analyzed lines after 8 days of salinity stress. **g–k** Nipponbare (Nipp), SLR1-OX, and SUMO1SLR1-OX plants under salt stress at the booting stage (80 mM) (*n* = 6). **g** Height as in aerial part length (*n* = 6). **h** Number of tillers (*n* = 6). **i** Panicle number (*n* > 10). **j** Percentage of filled seeds per total of seeds (*n* > 10) (g) 1000-grain weight (*n* > 10). Error bars correspond to standard error and statistical significance represented as * (*P* < 0.05) and **(*P* < 0.01) for comparing mock and stress conditions

Because DELLA protein accumulation was previously connected with salt stress tolerance (Achard et al. 2006), we assessed SLR1 protein levels in response to salt stress. Wild-type (WT) rice seedlings accumulated SLR1 protein in shoots during the first 8 h of stress (Fig. 3a). To assess SUMOylated SLR1 levels, we treated the protein extracts with recombinant SUMO ULP1, and we observed similar levels of SUMOylated SLR1 in both control and salt conditions.

To gain insight into the role of SLR1 SUMOylation in rice development and responses to salt stress, we generated transgenic lines overexpressing SLR1 (SLR1-OX), and lines constitutively expressing a mimetic form of the SUMOylated SLR1 (SUMO1SLR1-OX) resulting from the genetic fusion of mature SUMO1 to the K2 residue of SLR1 (Fig. 3b). The strategy of using SUMOylated mimetic isoforms originated by gene fusion with the SUMO coding sequence has been used in several biological systems, including plants (Ulrich 2008; Crozet et al. 2015). T0 plants with high SLR1-OX and SUMO1SLR1-OX expression levels bared no seeds, and T2 plants lost overexpression, showing the high regulation that DELLA proteins can suffer. For that reason, we could only work with T1 lines with less than 3.5-fold increases in SLR1 and SUMO1SLR1-OX protein levels (Fig. 3c). We confirmed the overexpression of non-SUMOylated SLR1 isoforms in both SLR1-OX and SUMO1SLR1, with the latter also showing a band with the expected mobility-shift of SLR1 corresponding to mono-SUMOylation (Fig. 3c). We also observed an increase in the amount of non-SUMOylated SLR1 protein in SUMO1SLR1-OX compared to WT and negative segregant (NS) lines, which could be arising from the processing by endogenous SUMO proteases because the fusion protein retains the SUMO diglycine protease cleavage motif (-GGK-) (Fig. 3c).

Rice is particularly sensitive to salt at both the seedling and booting stages (Gregorio et al. 1997). Therefore, we analyzed stress marker symptoms of SLR1-OX and SUMO1SLR1-OX lines subjected to high salt concentrations at the seedling stage, as well as yield parameters on salt treatments at the booting stage. Evaluation of symptoms in seedlings subjected to severe salinity (120 mM NaCl) for 8 days showed that both SUMO1SLR1-OX lines performed better under salt stress, displaying decreased symptom severity according to SES score (Standard Evaluation System for Rice) by IRRI (International Rice Research Institute) (Fig. 3d) (Gregorio et al. 1997), including minimal leaf tip rolling and increased survival (Fig. 3e–d). Plants overexpressing SUMO1SLR1 and SLR1-OX also recovered better than the wild-type Nipponbare in the 8 days following salt stress cessation (Fig. 3e).

Rice seedlings subjected to severe salinity showed the expected growth arrest in Nipponbare and the two independent SLR1-OX lines. This behavior was significantly attenuated in both SUMO1SLR1-OX lines. Under control conditions, SUMO1SLR1-OX lines also displayed some impairment in seedling growth, though this difference was not statistically significant compared to Nipponbare (Fig. 3f).

**Fig. 4:**
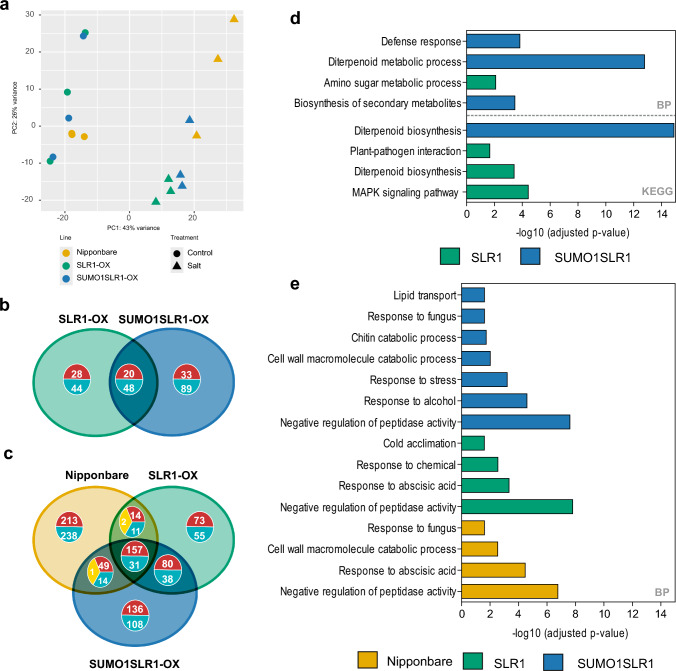
Salt treatment had a major contribution to the differences in gene expression among Nipponbare, SLR1-OX, and SUMO1SLR1-OX. **a** Principal Component Analysis (PCA) based on gene transcriptional profile obtained with RNA-seq of 14-day-old rice shoot samples from WT (Nipponbare), SLR1-OX, and SUMO1SLR1-OX lines, after 8 h under salt stress and control conditions. **b–c** Venn diagrams showing the overlaps between SLR1-OX and SUMO1SLR1-OX differentially expressed genes (DEGs) compared to Nipponbare in control conditions (**b**) and differentially expressed genes found in SLR1-OX, SUMO1SLR1-OX, and Nipponbare lines under salt stress compared to the respective genotypes under control conditions (**c**). Red: upregulated genes; blue: downregulated genes; yellow: contrasting gene expression. **d–e** GO functional enrichment analysis of the differently expressed genes in SLR1-OX and SUMO1SLR1-OX in control conditions (**d**), and SLR1-OX, SUMO1SLR1-OX, and Nipponbare lines under salt stress compared to the respective genotypes under control conditions (**e**), respectively. BP stands for biological processes

We next tested plant performance under salt stress at the booting stage, using panicle initiation as the cue for stress imposition. At this developmental stage, pollen viability is severely affected by high salinity (Negrão et al. 2011; Sarhadi et al. 2012). Upon measurement of agronomical parameters, we did not detect significant stress-induced changes in height (Fig. 4a), tillering, or panicle length when compared to control or among the genotypes (Fig. 3g–i). However, we observed that the number of filled grains and grain weight, both important parameters for addressing crop yield, were significantly improved in SUMO1SLR1-OX lines under salt stress. Both SLR1-OX and Nipponbare presented around a 10% decrease in filled grains when compared to mock conditions, while SUMO1SLR1-OX showed no differences in both salt and mock treatments (Fig. 3f, g). Although the SUMO1SLR1-OX lines had lower productivity under mock conditions compared to Nipponbare, they showed improved performance under salt stress. These results substantiate the significance of SUMOylation of SLR1 in salt stress tolerance acquisition and yield protection observed in two sensitive rice developmental stages.

### SLR1 SUMOylation regulates GA biosynthetic pathway transcripts in response to salt stress

Because DELLA proteins regulate gene expression, we exposed SLR1-OX, SUMO1SLR1-OX, and Nipponbare genetic background to high salt concentrations (120 mM NaCl) during 8 h at the seedling stage and collected samples for subsequent RNA-seq analysis (Table S3). We selected the lines SLR1-OX #2 and SUMO1SLR1-OX #2 for the RNA-seq assay because they showed similar non-SUMOylated SLR1 levels, thus isolating the effect of SLR1 SUMOylation. Before sending the samples for sequencing we assessed SLR1 transcript levels by RT-qPCR. Both lines exhibited a 1.8-fold increase in SLR1 transcript read counts when compared to Nipponbare (Fig. S3a). To confirm the efficacy of the stress treatment salt-responsive transcripts such as *Rab21* and *P5CS* were used as a transcriptional readout of the salt stress treatment. Both transcripts were highly increased during salt stress in all genotypes (Fig. S3b). Principal Component Analysis (PCA) revealed a clear separation of samples by treatment (Fig. 4a).

The constitutive overexpression of SLR1 and SUMO1SLR1 in control conditions resulted in 140 and 190 differentially expressed genes (DEGs), respectively, compared to WT (Fig. 4b, Tables S4.1 to S4.2). Functional annotation of SLR1-and SUMO1SLR1-OX DEGs in control conditions showed enrichment of gene ontology (GO) terms associated with diterpenoid biosynthesis (KEGG:00904) highlighting the impact of SLR1 on GA metabolism that appears to be exacerbated on SUMO1SLR1-OX line (Fig. 4d, Tables S4.1 and S4.2). Notably, in SUMO1SLR1-OX lines, we observed overall downregulation in genes coding for enzymes essential in non-gibberellin diterpenoid synthesis, namely syn-copalyl diphosphate synthase (CPS4) and ent-cassa-12,15-diene synthase (KSL7). These enzymes are part of a diterpenoid pathway branching point that feeds the phytoalexins biosynthetic pathway (Otomo et al. 2004). Concurrently, other genes related to phytoalexin and phytocassane synthesis, such as cytochrome P450 family members CYP701A8, CYP99A3, OsCYP76M7, CYP76M8, and CYP71Z7, were also downregulated in SUMO1SLR1-OX (Table S4.7). SLR1-OX shared a downregulation of CYP701A8, OsMAS, OsCYP76M7, and OsCYP99A3 with SUMO1SLR1-OX. Interestingly, SUMO1SLR1-OX also shows enriched GO terms related to defense response (GO:0006952) and biosynthesis of secondary metabolites (KEGG:01110).

Phenotypical discrimination of SLR1-OX and SUMO1SLR1-OX was observed under salt stress but not under control conditions at the seedling stage (Fig. 3e). Interestingly, a closer look into the distribution of SLR1-OX and SUMO1SLR1-OX samples in the PCA under control and stress conditions highlights a similar tendency (Fig. 4a). Hence, we focused on the analysis of SLR1-OX, SUMO1SLR1-OX, and Nipponbare lines under salt stress compared to the respective genotypes under control conditions. We observed that Nipponbare shows a higher number of DEGs (730) followed by SUMO1SLR1-OX (615) and SLR1 (461) (Fig. 4c, Tables S4.5.5 to S4.7). Looking at the GO terms enriched in all the genotypes, SUMO1SLR1-OX appears with unique biological processes such as lipid transport (GO:0006869) and chitin catabolic processes (GO:0006032).

Another interesting feature we observed in the SUMO1SLR1-OX lines, is the overexpression of YAB4 under control conditions, which mirrors the increase observed in Nipponbare under salt stress compared to its control. YAB4 is a well-known interactor of SLR1 and plays a key role in regulating the expression of GA20ox2 (Yang et al. 2016) (Tables S4.2 and S4.5).

### SUMOylation disrupts SLR1 interaction with stress-associated transcription factors

To further evaluate the molecular aspects of SUMO1SLR1-OX tolerance to salt stress, we explored the notion that SLR1 exerts its function through interaction with TFs and transcriptional regulators. We designed a strategy to predict SLR1 TF interactors that could explain, at least partially, the differences in gene expression observed in the transgenic lines upon exposure to salt stress. Briefly, we submitted the promoter sequences (1000 bp upstream to the Transcription Start Site) of exclusive SLR1- and SUMO1SLR1-dependent salt stress-responsive genes to an in silico search for TF binding sites (TFBSs) in the PlantPAN online database (Chow et al. 2016). With this approach, we identified 14 candidate TFs belonging to different families, including the TCP, bZIP, bHLH, and B3, which could be regulating the output gene expression of SLR1-OX and SUMO1SLR1-OX transgenic lines under salt stress (Table 1).

**Table 1:** Enriched binding sites and associated transcription factors in SLR1 DEGs promoters. Promoter sequence analysis output from PlantPAN with Support referencing the percentage of transcription factor binding elements (TFBS/Sequence) found in SLR1- and SUMO1SLR1-dependent stress-responsive DEGs

TFBS	Support	TF Family	TF	Annotation
GGTCCCAC	91.4	TCP	PCF1/2	essential for meristematic tissue-specificity expression of the PCNA gene promoter
TACGTA	91.4	(Others)	RISBZ3	found in seed storage protein gene promoters
GCACGTGC	94.7	bHLH	bHLH089/094	forms a ternary complex with RSS3 and TIFY11A/JAZ9 to negatively regulate jasmonate-responsive genes
ACACGTGT	97.4	bZIP	bZIP23/OSBZ8	binds specifically to the abiotic stress ABA-responsive elements (ABRE) in the promoter of target genes
TNCGTACAA	98	SBP	SPL1	trans-acting factor that binds specifically to the consensus nucleotide sequence 5’-TNCGTACAA-3’
TGAC	98.7	Homeobox	OSH1	involved in shoot formation during embryogenesis
CATGC	100	B3	IDEF1	involved in iron deficiency response and tolerance
CCAAT	100	NF-YB	NFBY1/2/3/4	component of the NF-Y/HAP transcription factor complex regulating the expression of photosynthetic genes

**Fig. 5:**
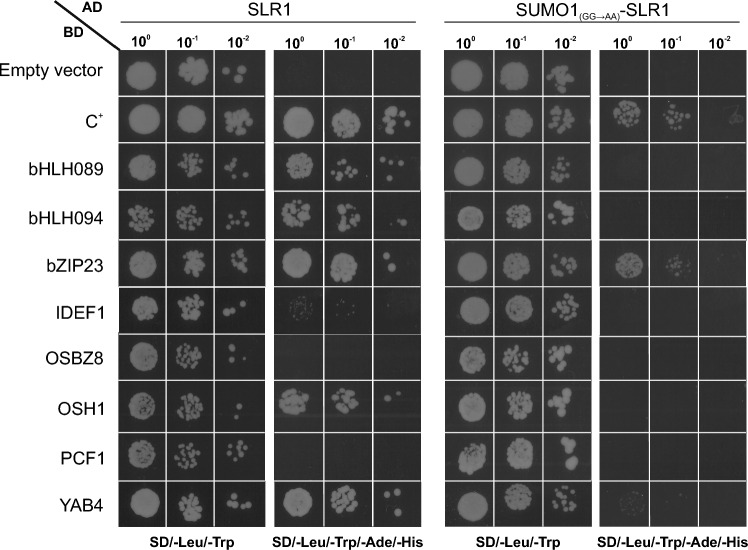
SLR1 SUMOylation impairs its interaction with key transcription factors. Y2H analysis of both SLR1 and SUMO1_(GG-AA)_-SLR1. SUMO1-SLR1 fusion carries two mutations in the SUMO-protease cleavage motif (GG → AA) to avoid its removal by endogenous yeast proteases and guarantee constitutive SUMO attachment to SLR1 during the assay SLR1 and SUMO1_(GG→AA)_-SLR1 were fused with the GAL4 activation domain (AD) and bHLH089, bHLH094, bZIP23, IDEF1, OSBZ8, OSH1, PCF1, and YAB4 with the GAL4 DNA binding domain (BD) into appropriate expression vectors before transfer into yeast (Y2HGold strain). The different yeast strains were plated on a synthetic complete selective medium lacking Leu and Trp (SD/-Leu/-Trp) or on a synthetic complete medium lacking Trp, Leu, Ade, and His (SD/-Leu/-Trp/-Ade/-His) for the screening. pAD-WT/pBD-WT (Wild-type fragment C of lambda cI repressor) was used as a positive control (C +). The empty BD vector was used as a negative control (Fig. S5a). The interaction was confirmed by three different clones

The interactions between SLR1 and 7 candidate TF interactors (bHLH089, bHLH094, bZIP23, IDEF1, OSBZ8, OSH1, and PCF1) and their potential regulation by SUMOylation were further assessed through Y2H assays and BiFC analyses (Fig. 5, Fig. S4 and S5). We used YAB4 as a positive control since it is a well-established interactor of SLR1 (Yang et al. 2016) but also due to its high expression level in SUMO1SLR1-OX in control conditions. Interestingly, both techniques confirmed bZIP23, bHLH089, bHLH094, and OSH1 as SLR1 interactors. These interactions were disrupted by the presence of SUMOylated SLR1 (SUMO1_(GG-AA)_-SLR1), suggesting regulation by SUMOylation (Fig. 5 and Fig. S4). Neither IDEF1, OSBZ8 nor PCF1 showed interactions with SLR1 or SUMO1_(GG-AA)_-SLR1.

## Discussion

DELLA proteins are central regulators of plant productivity and stress responses, and the hormonal crosstalk mediated by these proteins is essential for plant plasticity to endure stress. As other hormone modulators, DELLA activity is regulated by post-translational modifications, such as ubiquitination, phosphorylation, *O*-fucosylation, *O*-GlcNAcylation, and SUMOylation (Itoh et al. 2003; Dai and Xue 2010; Conti et al. 2014; Zentella et al. 2016, 2017; Yano et al. 2019). Recently, SUMO proteases were linked to salt stress response in rice (Srivastava et al. 2016, 2017). Although deSUMOylation was proposed as a general mechanism of tolerance acquisition in response to salt, we show that artificially SUMOylated SLR1 could alleviate the salt stress impact on rice productivity, also improving recovery after stress imposition is released.

DELLA proteins control several plant processes through interaction with a myriad of transcriptional factors (Van De Velde et al. 2017). By affecting protein–protein interaction, SLR1 SUMOylation has the potential to modulate plant processes by altering gene expression dependent upon SLR1-TFs interactions. Indeed, transcriptomic analysis of SUMOylated SLR1 overexpressing lines suggested that the modified SLR1 plays a role in early transcriptional responses to salt stress, affecting the expression of key genes coding for enzymes controlling GA biosynthesis. We determined that SLR1 SUMOylation preferentially occurs at lysine 2 residue (Fig. 1a–c) under the tested in vitro conditions. However, we were unable to confirm that K2 is also SUMOylated in rice in vivo conditions, which is not unusual since SUMOylated forms of a protein are normally of low abundance. Our in silico SUMOylation prediction, coupled with a phylogenetic analysis, revealed that both K2 and K65 are conserved SUMOylation sites and are promising candidates for in vivo SUMOylation. Both K2 and K65 are located in the DELLA domain, opening the possibility that they may have similar roles in regulating DELLA proteins. Supporting this, we show that SLR1 SUMOylation at K2 impacts salt stress tolerance, as suggested for RGA K65 SUMO modification in Arabidopsis (Conti et al. 2014). Furthermore, we observed an increase in non-SUMOylated SLR1 in the SUMO1SLR1-OX lines that we attribute to the cleavage of the SUMO *in planta*. Yet, we cannot exclude that native non-SUMOylated SLR1 can be stabilized by the presence of extra SUMO-SLR1 in transgenic lines, as previously shown by Conti et al. (2014).

SUMOylation of specific targets was suggested to be crucial for stress tolerance, fertility, and yield maintenance in rice. For instance, bZIP23 SUMOylation levels are modulated by OTS1, which affects its stability and the plant’s ability to deal with drought and salt stress (Srivastava et al. 2017). Interestingly, we found that bZIP23 interacts with SLR1, and the presence of SUMOylation disrupted that interaction. This is also the case of bHLH089 and bHLH094, which are novel SLR1 interactors. These two bHLHs were previously implicated in the jasmonic acid (JA) signaling pathway (Toda et al. 2013). Furthermore, our in silico promoter analysis highlighted bHLH089 and bHLH094 as candidate regulators of JAMYB, which are relevant in the JA signaling pathway. Yet, the specific role of bHLH089 and bHLH094 in stress response and plant development is still largely unknown.

It has been previously shown that YAB4 interacts with SLR1 to repress *GA20ox2* gene expression and restrain development in rice (Yang et al. 2016), and we confirmed the interaction between SLR1 and YAB4 by Y2H and BiFC assays. We additionally showed that SUMOylation of SLR1 abolished its interaction with YAB4. Curiously, *YAB4* levels are induced on the SUMO1SLR1-OX line. Considering that both lines, SLR1-OX and SUMO1SLR1-OX, have similar non-modified SLR1 levels, this suggests that the upregulation of *YAB4* is due to the presence of SUMOylated SLR1. In this sense, we can hypothesize that SUMO1SLR1 is disrupting SLR1-*YAB4* interaction (Fig. 5, Fig. S4) to regulate the, previously suggested effect of YAB4 on SLR1 levels (Yang et al. 2016).

Finally, we also observed the disruption of the interaction of SLR1 with OSH1, a KNOTTED1-like homeobox protein, by SLR1 SUMOylation. OSH1 represses *GA20ox2* expression (Sakamoto et al. 2006), suggesting another potential point of cross-regulation between SLR1 levels and GA biosynthesis.

Overall, we hypothesize that SUMOylation-mediated disruption of SLR1 interactions can be a rapid response mechanism during specific stimuli, to prevent or abolish binding to transcription factors, thereby modulating downstream genes. The emergence of SUMOylation of SLR1 as an effector of rice tolerance to salt stress, through the growth-maintaining modulation of protein–protein interactions with transcriptional regulators such as YAB4, is evidence of the tight regulation of the GA pathway by rice DELLA during stress. Recent studies demonstrated that DELLA proteins are targets of different post-translational modifications, which could dynamically adjust their interactions with transcriptional regulators, ensuring the best developmental decisions when facing stress. Further studies must be performed to unveil upstream effectors that perceive stress and the underlying signal transduction pathway that leads to SLR1 SUMOylation. By establishing the trigger for SLR1 SUMOylation and its effect on stress-induced SLR1 protein–protein interactions, new strategies can be developed involving the transient post-translational modification of DELLA and selection of growth-favorable SLR1 alleles. This can be used to activate the SUMO-regulated tolerance mechanism when stress is imminent and protect crops from yield losses due to abiotic stress.

## Supplementary Information

Below is the link to the electronic supplementary material.

Supplementary file1 (PDF 22 KB)

Supplementary file2 (DOCX 4490 KB)

Supplementary file3 (XLSX 100 KB)

Supplementary file4 (DOCX 25 KB)

Supplementary file5 (XLSX 5299 KB)

## Data Availability

Data publicly available in a repository: RNA-Seq data were deposited into BioStudies data repository under accession number E-MTAB-14289 and are available at the following URL: https://www.ebi.ac.uk/biostudies/arrayexpress/studies/E-MTAB-14289.

## Abbreviations

GA: Gibberellin
PAC: Paclobutrazol
RGA: REPRESSOR OF GA
TF: Transcription factor
