# Supplementary material for: SUMOylation of rice DELLA SLR1 modulates transcriptional responses and improves yield under salt stress
Source: Planta. 2024 Nov 8;260(6):136. doi: 10.1007/s00425-024-04565-1 (PMC11549141; doi:10.1007/s00425-024-04565-1)
Supplement: Supplementary file 2 — Supplementary file2 (DOCX 4490 KB) [file 425_2024_4565_MOESM2_ESM.docx]

**Title:**

**SUMOylation of rice DELLA SLR1 modulates transcriptional responses and improves yield under salt stress.**

**Journal Name:**

Planta

**Authors:**

Telma Fernandes^*^, Nuno M. Gonçalves^*^, Cleverson C. Matiolli, Mafalda A. A. Rodrigues, Pedro M. Barros, M. Margarida Oliveira, Isabel A. Abreu^1^

**Affiliations:**

Instituto de Tecnologia Química e Biológica, Universidade Nova de Lisboa (ITQB NOVA), 2780-157, Oeiras, Portugal

**Corresponding author E-mail:**

[abreu@itqb.unl.pt](mailto:abreu@itqb.unl.pt)

**Supplemental Figures**

**[Please see FigS1.pdf file]**

**Fig. S1** Phylogenetic analysis of DELLA proteins. The phylogenetic reconstruction includes DELLA orthologs found in representative plant taxa, excluding DELLA 3 sequences. Sequences were aligned with the MAFFT algorithm, and maximum likelihood trees were inferred using the PROTCAT model, JTT matrix, and 1000 bootstrap replications in RaxML v8.2.12. The consensus-rooted tree was visualized and annotated using the FigTree software. The numbers on each branch represent the percentages of bootstrap, and the colors represent the different taxa: Pink, Angiosperm-Eudicots; Blue, Angiosperm-Monocots; Yellow, Angiosperms-Magnoliales; Grey, Angiosperms-Amborellale; Green, Gymnosperms; Dark yellow, Pteridophytes; Orange, Bryophytes.


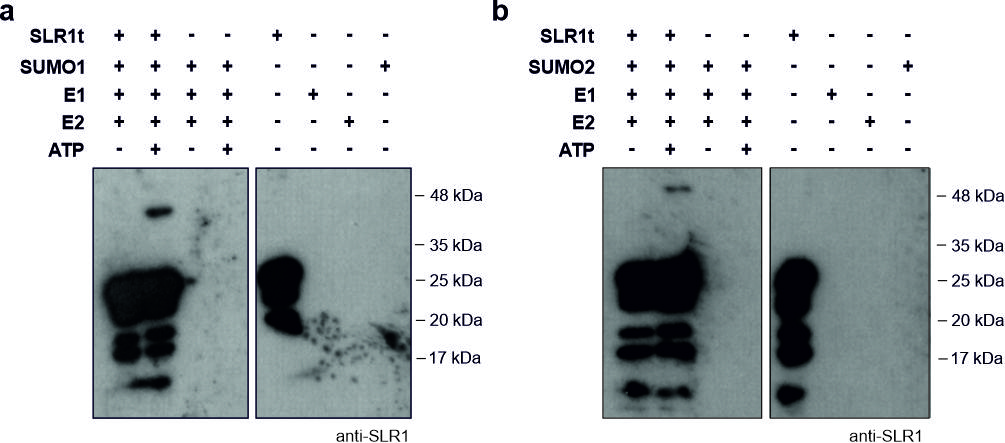


**Fig. S2** SLR1 can be SUMOylated by both SUMO1 and SUMO2 *in vitro*. SUMOylation assays with and without ATP using SLR1t recombinant protein as detected by immunoblot using custom-made anti-SLR1. **a** N-terminal truncated SLR1t and SUMO1. **b** N-terminal truncated SLR1t and SUMO2.


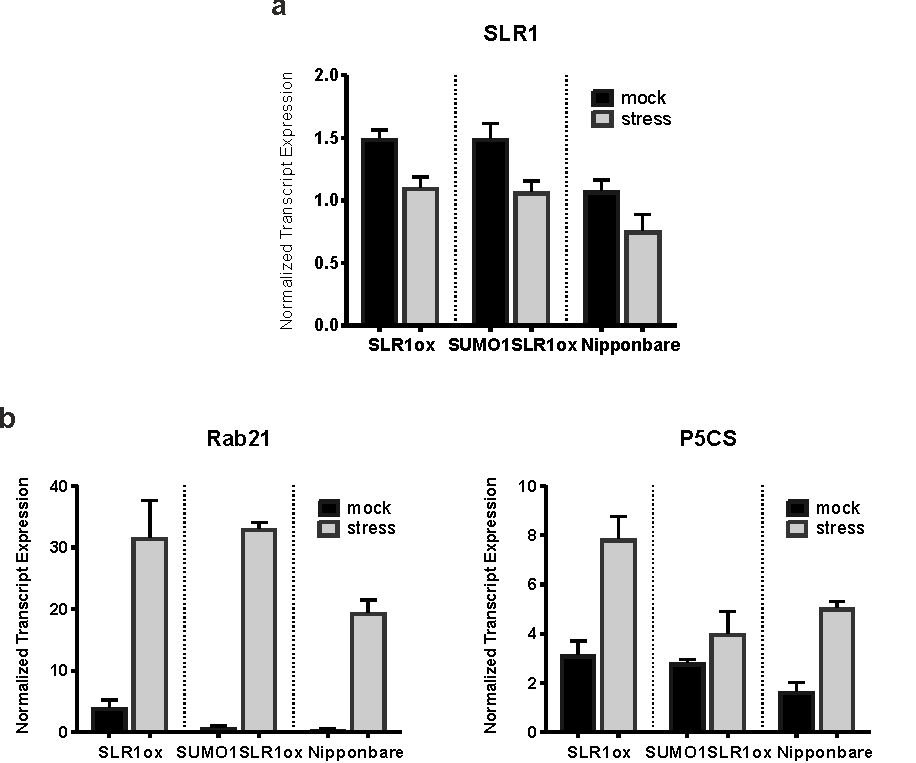


**Fig. S3** RNA-seq sample evaluation of 14 DAG shoot samples for SLR1-OX, SUMO1SLR1-OX, and wild-type background (Nipponbare) upon salinity imposition with 120 mM NaCl in hydroponics. **a-b** RT-qPCR Transcript expression analysis of selected rice genes *SLR1, Rab21* and *P5CS*, in shoots after 8 hours of salinity stress imposition (stress) along with untreated control (mock). Individual gene expression normalized to two housekeeping genes (*UBC2* and *UBQ10*).


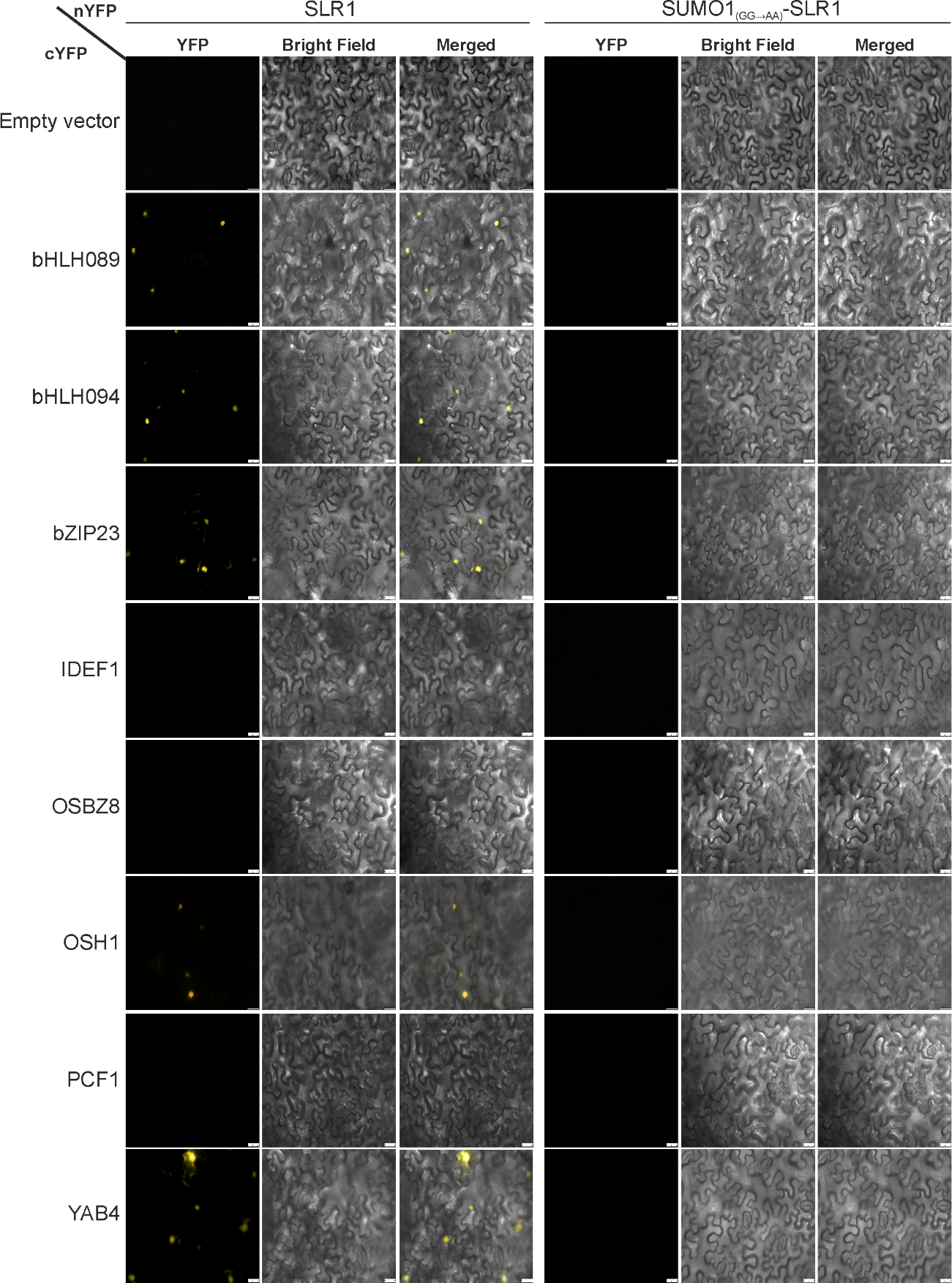


**Fig. S4** Validation of interaction between SLR1 and SUMO1_(GG→AA)_-SLR1 with the previously identified TFs. BiFC assay where SLR1 and SUMO1_(GG→AA)_-SLR1 was fused with the N-terminal part of YFP (nYFP) and bHLH089, bHLH094, bZIP23, IDEF1, OSBZ8, OSH1, PCF1 and YAB4 with the C-terminal part of YFP (cYFP) into appropriate expression vectors before Agrobacterium transfection of *N. benthamiana* leaves and analysis by fluorescence microscopy. bHLH089, bHLH094, bZIP23, IDEF1, OSBZ8, OSH1, PCF1, and YAB4 with the C-terminal part of YFP (cYFP) against nYFP alone was used as negative control (Fig. S5b). Bars = 25 µm.


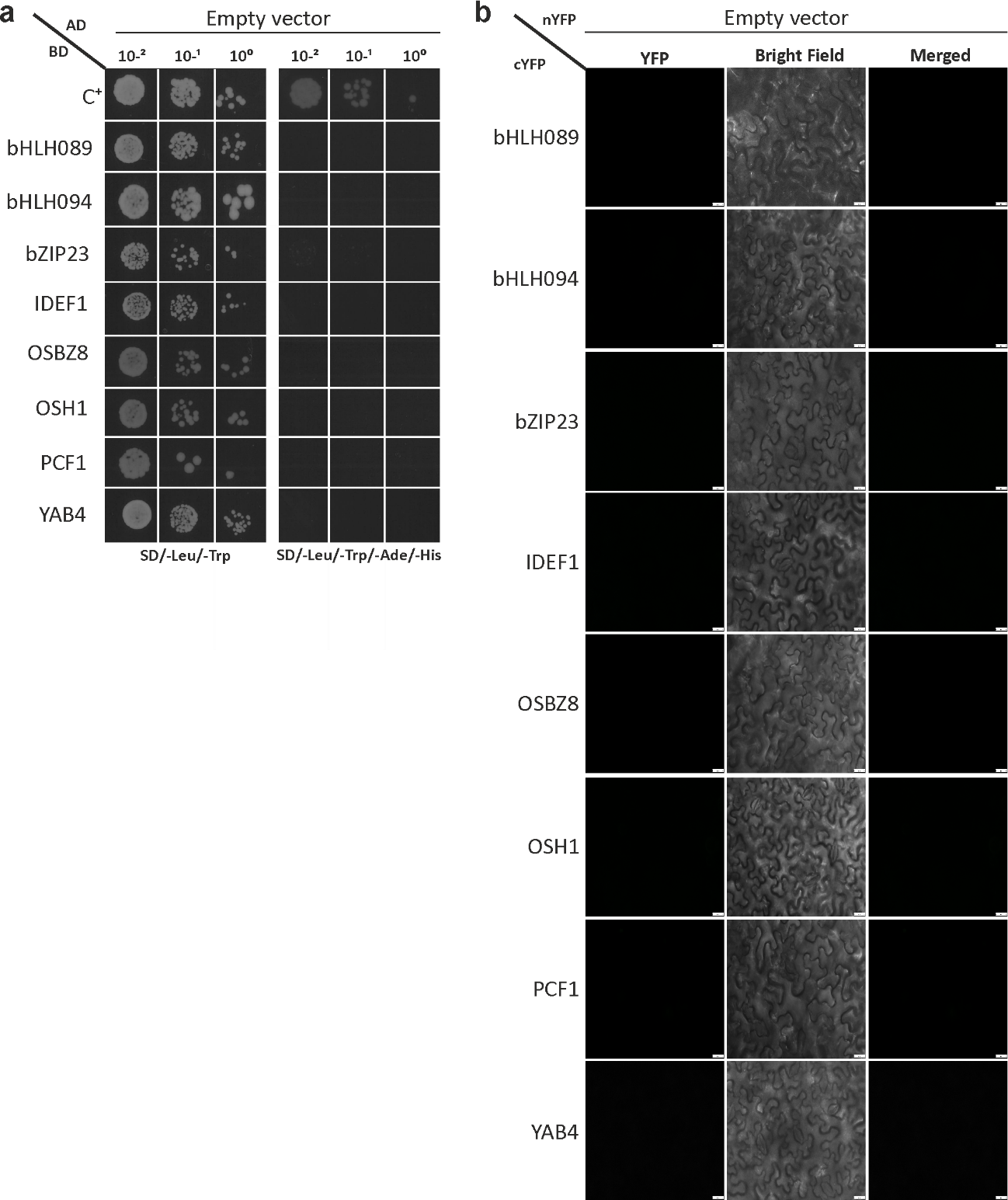


**Fig. S5** Protein-protein interaction negative controls for Y2H and BiFC assays (Fig. 5 and Fig. S4). **a** Yeast two-hybrid assay with the activation domain not interacting with bHLH089, bHLH094, bZIP23, IDEF1, OSBZ8, OSH1, PCF1, and YAB4. The different yeast strains were plated on a synthetic complete selective medium lacking Leu and Trp (SD/-Leu/-Trp) or on a synthetic complete medium lacking Trp, Leu, Ade, and His (SD/-Leu/-Trp/-Ade/-His) for the screening. pAD-WT/pBD-WT (Wild-type fragment C of lambda cI repressor) was used as positive control (C+) **b** BiFC assay testing YFN43 (nYFP) empty vector for interaction with cYFP (YFC43) fused to bHLH089, bHLH094, bZIP23, IDEF1, OSBZ8, OSH1, PCF1 and YAB4.
