# Supplementary material for: SUMOylation of rice DELLA SLR1 modulates transcriptional responses and improves yield under salt stress
Source: Planta. 2024 Nov 8;260(6):136. doi: 10.1007/s00425-024-04565-1 (PMC11549141; doi:10.1007/s00425-024-04565-1)
Supplement: Supplementary file 4 — Supplementary file4 (DOCX 25 KB) [file 425_2024_4565_MOESM4_ESM.docx]

**Title:**

**SUMOylation of rice DELLA SLR1 modulates transcriptional responses and improves yield under salt stress.**

**Journal Name:**

Planta

**Authors:**

Telma Fernandes^*^, Nuno M. Gonçalves^*^, Cleverson C. Matiolli, Mafalda A. A. Rodrigues, Pedro M. Barros, M. Margarida Oliveira, Isabel A. Abreu^1^

**Affiliations:**

Instituto de Tecnologia Química e Biológica, Universidade Nova de Lisboa (ITQB NOVA), 2780-157, Oeiras, Portugal

**Corresponding author E-mail:**

[abreu@itqb.unl.pt](mailto:abreu@itqb.unl.pt)

**Supplemental Tables**

**[Please see Supplementary Table 1.xlsx file]**

**Table S1 High-probability SUMOylation sites identified by GPS-SUMO in 119 species representatives of several important taxa.** DELLA protein sequences were retrieved from Plaza (Monocots 5.0; Dicots 5.0; https://bioinformatics.psb.ugent.be/plaza/) using SLR1 and AtRGA query terms for gene family identification, Phytozome (https://phytozome-next.jgi.doe.gov/) and NCBI genome (https://www.ncbi.nlm.nih.gov/genome/). These datasets comprise 85 species for dicots and 27 monocots, bryophytes (*Physcomitrium* *patens* and *Marchantia polymorpha*), Pteridophytes (*Ceratopteris richardii* and *Selaginella moellendorffii*), and Gymnosperms (*Sequoiadendron giganteum, Thuja plicata*, and *Taxus chinensis*) and the evolutionary important species such as *Amborella trichopoda*, *Spirodela polyrhiza* and *Zoostera marina*. The DELLA sequences lacking the N-terminal DELLA regulatory domain from Angiosperms and Gymnosperms (DELLA 3) were removed.

**Table S2 List of primers used in this study.**

| Primers | Sequence |
| --- | --- |
| OsSLR1-GW-Fw | GGGGACAAGTTTGTACAAAAAAGCAGGCTTAATGAAGCGCGAGTACCAAGA |
| OsSLR1-GW-Rv | GGGGACCACTTTGTACAAGAAAGCTGGGTTAGCGTCCCAAAAACTTTGC |
| OsSLR1-CDS-Fw | ATGAAGCGCGAGTACCAAGA |
| OsSLR1-CDS-Rv | AGCGTCCCAAAAACTTTGC |
| pET-Fw-EcoRI | ATAGAATTCATGAAGCGCGAGTACCAAGA |
| pET-Rv-PspOMI | ATATGGGCCCAGCGTCCCAAAAACTTTGC |
| SLR1t-STOP-Fw | GGCAGCACGTCGTAATCCTCATCGTCG |
| SLR1t-STOP-Rv | CGACGATGAGGATTACGACGTGCTGCC |
| SLR1t-K2R-Fw | GTACTCGCGCCTCATGAATTCGGATCCGC |
| SLR1t-K2R-Rv | CGCGGATCCGAATTCATGAGGCGCGAGTAC |
| SLR1t-K60R-Fw | GCTCCAGCCTCTGCGCGACGTCGG |
| SLR1t-K60R-Rv | CCGACGTCGCGCAGAGGCTGGAGC |
| OL-SLR1+SUMO1-3'-Fw | CGGGGACGAGATCGACGCCATGCTCCACCAGACTGGAGGCAAGCGCGAGTACCAAGAAGC |
| OL-SUMO1+SLR1-5'-Rv | CGCCGCCGCTGCTCCCGCCGGCTTCTTGGTACTCGCGCTTGCCTCCAGTCTGGTGGAGCAT |
| OsSUMO1-GW-Fw | GGGGACAAGTTTGTACAAAAAAGCAGGCTTAATGTCGGCCGCCGGGGAGGAGGA |
| Rht-like-GW-FW | GGGGACAAGTTTGTACAAAAAAGCAGGCTTAATGGGGATGGGCGGCGTGA |
| OsSLR1-Fw | GATCGGGCTTACGGTTCTCG |
| OsSLR1-Rv | GCTAGGAGGACCAAGGAACG |
| OsUBC2-Fw | TTGCATTCTCTATTCCTGAGCA |
| OsUBC2-Rv | CAGGCAAATCTCACCTGTCTT |
| OsUBQ10-Fw | TGGTCAGTAATCAGCCAGTTTGG |
| OsUBQ10-Rv | GCACCACAAATACTTGACGAACAG |

**Table S3 - RNA-seq read count for each of the sequenced libraries.**

| Treatment | Genotypes | High quality reads | Uniquely Mapped Reads | Assigned to features |
| --- | --- | --- | --- | --- |
| Mock | Nipponbare 1 | 26327856 | 22116060 | 17342854 |
|  | Nipponbare 2 | 22760630 | 18960832 | 14778380 |
|  | Nipponbare 3 | 29327311 | 24011061 | 18907046 |
| Salt | Nipponbare 1 | 21333054 | 17937376 | 14196444 |
|  | Nipponbare 2 | 25928265 | 22386888 | 17795044 |
|  | Nipponbare 3 | 29243298 | 25097215 | 19959516 |
| Mock | SUMO1SLR1-OX 1 | 24595809 | 20723581 | 16428190 |
|  | SUMO1SLR1-OX 2 | 15688568 | 13193609 | 10244357 |
|  | SUMO1SLR1-OX 3 | 28844447 | 24327558 | 18819989 |
| Salt | SUMO1SLR1-OX 1 | 15736987 | 12915106 | 10326458 |
|  | SUMO1SLR1-OX 2 | 17789610 | 13402412 | 10635839 |
|  | SUMO1SLR1-OX 3 | 35017712 | 29606468 | 23616327 |
| Mock | SLR1-OX 1 | 30716518 | 25878098 | 20202963 |
|  | SLR1-OX 2 | 13890481 | 11882981 | 9300527 |
|  | SLR1-OX 3 | 26433011 | 22621127 | 17886157 |
| Salt | SLR1-OX 1 | 34447937 | 28967172 | 23194835 |
|  | SLR1-OX 2 | 29929082 | 25447116 | 20557596 |
|  | SLR1-OX 3 | 24588748 | 20208688 | 16117452 |

**[Please see Supplementary Table 4.xlsx file]**

**Table S4 - RNA seq data showing >1.5 log2 fold change differentially expressed genes SLR1-OX and SUMO1SLR1-OX compared to Nipponbare in control and salt conditions and, Nipponbare, SLR1-OX and SUMOSLR1-OX in salt stress compared to control conditions.**
