# Supplementary material for: SUMOylation of rice DELLA SLR1 modulates transcriptional responses and improves yield under salt stress
Source: Planta. 2024 Nov 8;260(6):136. doi: 10.1007/s00425-024-04565-1 (PMC11549141; doi:10.1007/s00425-024-04565-1)

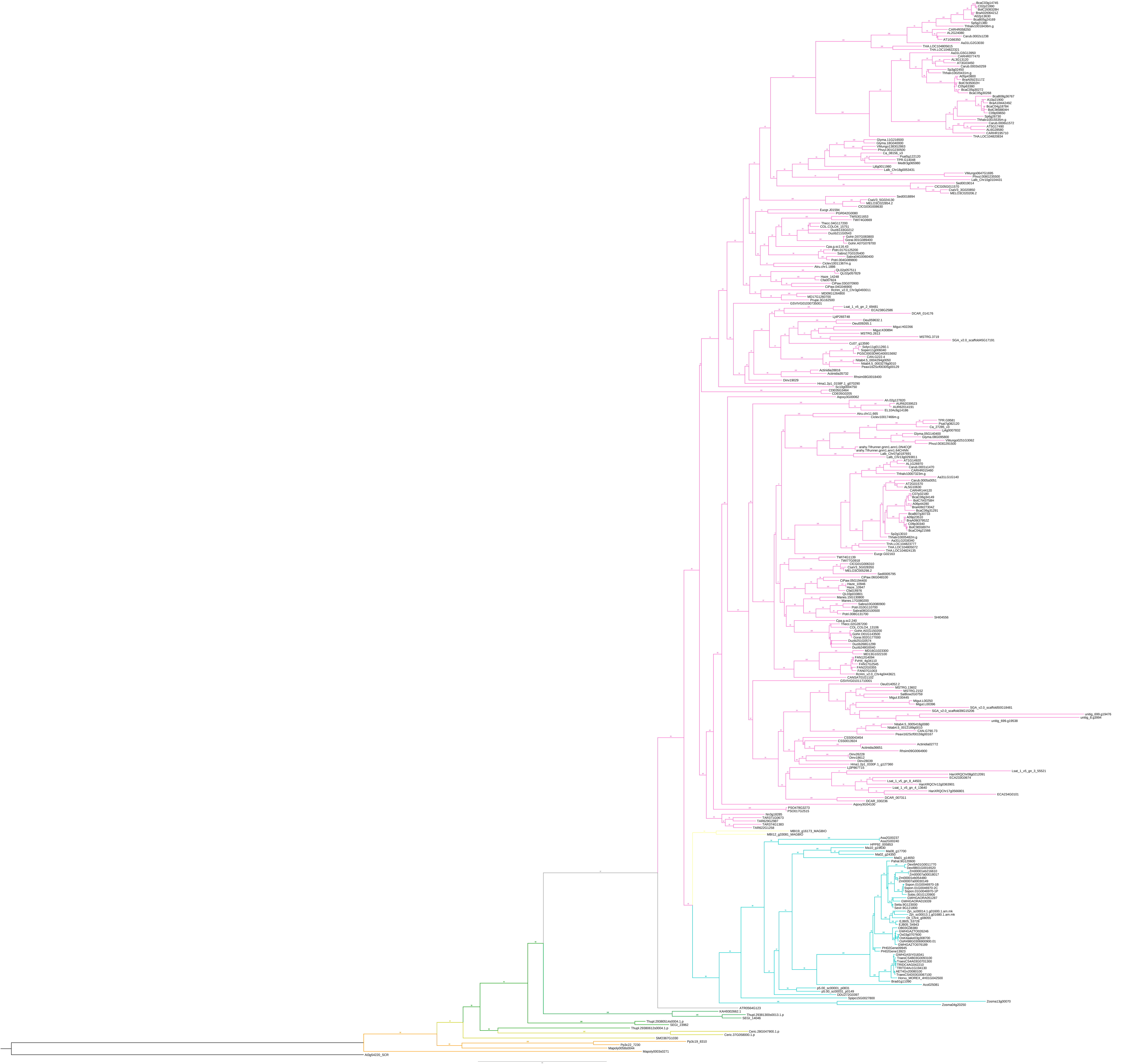

Supplement: Supplementary file 1 — Supplementary file1 (PDF 22 KB) [file 425_2024_4565_MOESM1_ESM.pdf]
